# Intensive Care Unit Acquired Weakness as a Modifiable Organ Dysfunction? A Narrative Review of Evolving Diagnostic and Therapeutic Concepts

**DOI:** 10.3390/nu18050820

**Published:** 2026-03-03

**Authors:** Moritz L. Schmidbauer, Konstantinos Dimitriadis

**Affiliations:** Department of Neurology, University Hospital LMU Munich, Marchioninistr. 15, 81377 Munich, Germany

**Keywords:** intensive care unit acquired weakness, sarcopenia, post-intensive care syndrome, critical illness myopathy, critical illness polyneuropathy, critical illness polyneuromyopathy

## Abstract

Intensive Care Unit Acquired Weakness (ICUAW) is a highly prevalent neuromuscular complication affecting around 40% of critically ill patients, rising to over 80% in high-risk cohorts. It is independently associated with prolonged mechanical ventilation, increased intensive care unit (ICU) and hospital length of stay, elevated mortality (in-hospital, 1-year, and 5-year), higher healthcare costs, and long-term functional impairment. ICUAW is clinically defined by symmetric flaccid tetraparesis, frequently involving respiratory muscles, and exhibits significant pathobiological heterogeneity. Further subclassification is based on neurotopographic patterns: Critical Illness Polyneuropathy (CIP), Myopathy (CIM), and Polyneuromyopathy (CIPNM). Diagnosis typically relies on the Medical Research Council (MRC) Sum Score, with a threshold of <48 indicating clinically relevant weakness. While adjunct modalities such as electromyography/nerve conduction studies support assessment, their utility may be limited by patient cooperation and availability. Preventive strategies center on modifiable metabolic factors. Caloric and protein deficits exacerbate catabolism, while overfeeding—linked to anabolic resistance and stress hyperglycemia—also impairs recovery. To date, pharmacologic interventions remain inconclusive. However, early mobilization and neuromuscular electrical stimulation are promising non-pharmacologic strategies. The multifactorial and heterogeneous pathophysiology of ICUAW highlights the need for a biologically refined definition that can guide future targeted therapeutic interventions. Comprehensive multimodal strategies, together with structured long-term follow-up in Post-Intensive Care Syndrome (PICS) clinics, are essential for improving outcomes in this prevalent complication of critical care.

## 1. Background

**Figure 1:**
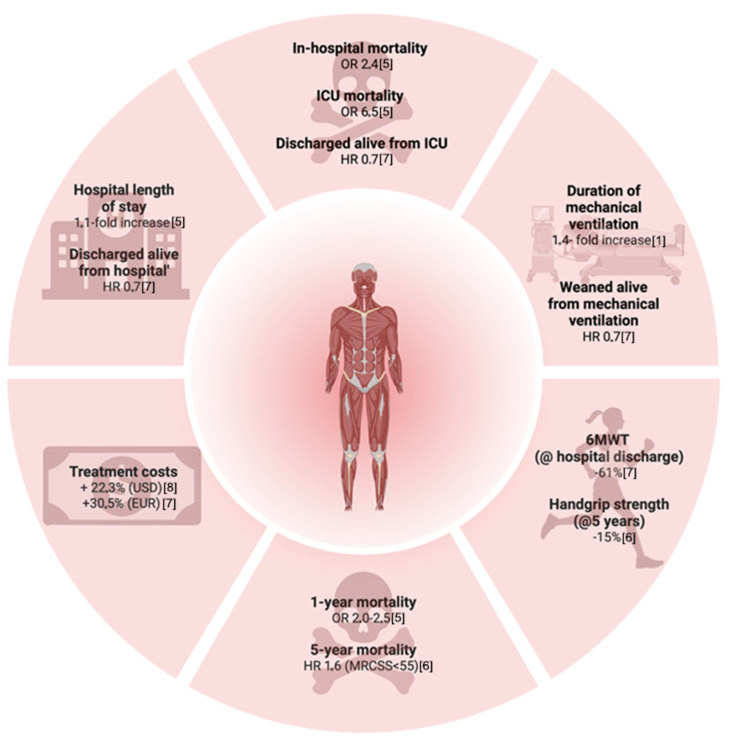
Outcomes associated with ICUAW. ICU—Intensive Care Unit; HR—Hazard Ratio; USD—US Dollar; EUR—Euro; OR—Odds Ratio; MRCSS—Medical Research Council Sum Score; 6MWT—6 Minute Walk Test. Created with Biorender.com.

Intensive Care Unit Acquired Weakness (ICUAW) is a clinical syndrome characterized by an acquired neuromuscular dysfunction secondary to critical illness and its associated treatments. The reported prevalence of this complication in the intensive care setting varies substantially due to methodological heterogeneity and differences in patient populations. Nevertheless, with a median prevalence of 43% (and up to 80% of patients affected in high-risk cohorts, e.g., sepsis), ICUAW is ubiquitous in critical care [1,2,3,4]. Moreover, ICUAW has been independently associated with prolonged mechanical ventilation [1], extended hospital length of stay [5], increased mortality (during hospitalization, at 1-year, and 5-year follow-up) [5,6,7], elevated healthcare costs [7,8], and persistent functional impairment lasting up to five years following ICU discharge (Figure 1) [6,7]. The combination of high prevalence and profound, long-term negative consequences underscores the clinical significance of this condition. Accordingly, ICUAW is now recognized as a core component of the Post-Intensive Care Syndrome (PICS), reflecting its critical role in the continuum of recovery from critical illness [9].

Several recent reviews have provided valuable updates on the epidemiology, risk factors, diagnostic strategies, and management of ICUAW [4,10,11,12,13,14]. Building on this foundation, the present review adopts a neurologically oriented syndromic approach that integrates clinical presentation with neurotopographic diagnoses and underlying pathophysiological mechanisms. In parallel, we summarize recent therapeutic evidence and frame preventive and treatment strategies within this mechanism-based perspective.

## 2. Methods

This narrative review was conducted to synthesize and interpret emerging evidence across clinical, pathophysiological, and therapeutic domains of ICUAW. A structured, non-systematic search of PubMed/MEDLINE and the Cochrane Library was performed for English-language publications up to December 2025 using combinations of MeSH terms and free-text keywords (including “Muscle Weakness,” “ICUAW”, “Critical Illness Polyneuropathy,” and “Critical Illness Myopathy”). The scope was focused on studies addressing pathophysiology as well as diagnostic and therapeutic strategies. Priority was given to recent systematic reviews and randomized controlled trials. Additional relevant publications were identified through reference screening of key articles. Literature inclusion was guided by thematic relevance and conceptual contribution.

## 3. Clinical, Neurotopographic, and Pathophysiological Dimensions of ICUAW

**Figure 2:**
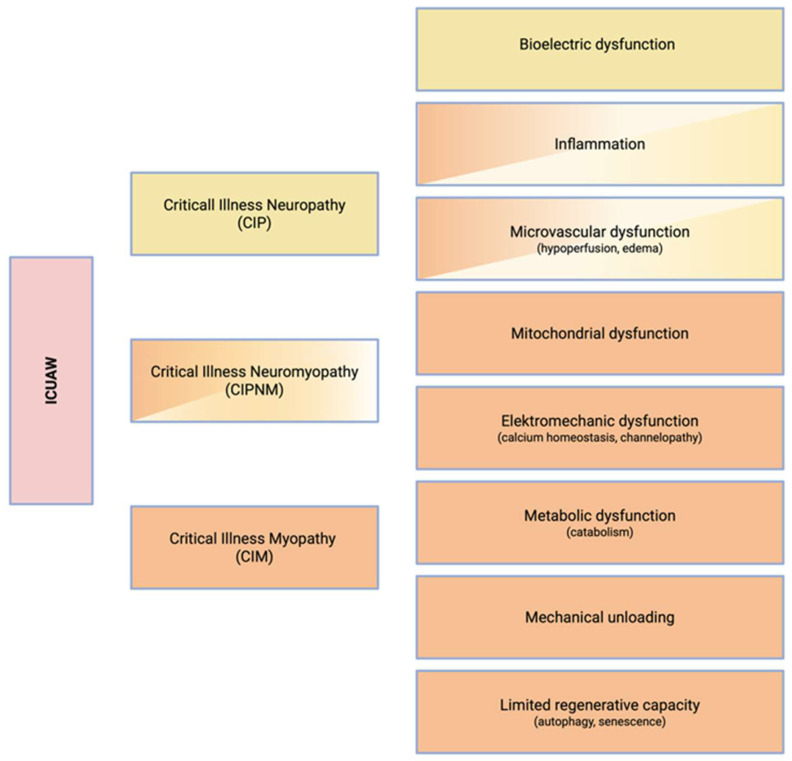
Clinical, neurotopographic and pathophysiological dimensions of ICUAW. The clinical syndromic diagnosis of generalized muscle weakness (first column; ICUAW) can only be sub-classified neurotopographically (second column; CIP, CIM, or CIPNM) through the additional use of instrumental diagnostics. At the level of pathophysiology (third column), there is also an overlap in key mechanisms of injury (inflammation, microvascular dysfunction, mitochondrial dysfunction). Created with Biorender.com.

ICUAW is primarily defined as a clinical syndrome characterized by symmetric, flaccid tetraparesis with relative sparing of cranial nerve-innervated musculature (first column, Figure 2). However, the respiratory as well as the pharyngeal musculature may also be affected [15]. Hence, dysphagia and failed weaning from mechanical ventilation should raise clinical suspicion for ICUAW, even in the absence of overt limb involvement.

To better reflect the full spectrum of this condition and to decouple the diagnosis from intensive care treatment per se, international experts have recently proposed the terms Critical Illness Weakness (CIW) and Critical Illness Associated Diaphragmatic Weakness (CIDW) as alternative nomenclature [15,16]. However, ICUAW/CIW/CIDW are all clinical classifications. Thus, these classifications remain limited to the syndromic level and cannot adequately account for the substantial heterogeneity and often overlapping pathologies [13,14].

As a result, further subclassification is frequently employed in the literature based on the neurotopographic characteristics (second column, Figure 2): Critical Illness Polyneuropathy (CIP), Critical Illness Myopathy (CIM), and Critical Illness Polyneuromyopathy (CIPNM)—the latter reflecting the coexistence of both neuropathic and myopathic patterns [17]. It is important to note that such an anatomical subclassification requires the use of advanced diagnostic modalities (see section below). In the absence of such investigations, retaining the syndromic diagnosis of ICUAW remains both pragmatic and clinically appropriate. Classification along the pathophysiological axis is often limited by the insufficient diagnostic resolution of tools routinely available in clinical practice (third column, Figure 2).

## 4. Pathophysiology

ICUAW (and its neurotopographically defined sub-entities CIP/CIM and CIPNM) involves a complex interplay of biological mechanisms in response to critical illness and invasive treatment in the ICU, resulting in a multifaceted pathology of the neuromuscular system (Figure 2).

### 4.1. Myopathy

The underlying pathophysiological mechanisms of myopathy can be broadly classified into atrophic and dysfunctional processes, which form the conceptual framework for the subsequent sections.

Muscle atrophy in critical illness results from an imbalance in protein turnover, with degradation exceeding synthesis [4,13,14]. This catabolic state is driven by the systemic stress response, characterized by elevated cortisol levels and reduced availability of anabolic hormones such as insulin and IGF-1 [18]. A central pathway mediating proteolysis is the ubiquitin–proteasome system (UPS), which is consistently upregulated during the acute phase of critical illness [13]. However, emerging evidence suggests that complete suppression of UPS activity may be detrimental, as transient activation appears to be part of an adaptive response. In ICU survivors, UPS activation is typically observed early after discharge but resolves within months [19]. Experimental inhibition of the UPS with bortezomib reduces hypercatabolism in animal models, but is associated with increased mortality, highlighting the complexity of this pathway [20].

Importantly, muscle tissue during critical illness exhibits anabolic resistance, meaning that it responds poorly to nutritional stimuli intended to stimulate protein synthesis. Consequently, increased nutrient provision alone does not reverse hypercatabolism. Autophagy represents another key regulatory mechanism. While controlled autophagy is essential for clearing damaged cellular components in the acute phase, excessive or dysregulated autophagy during recovery may contribute to sustained muscle loss [21,22]. Consistent with this concept, early aggressive feeding has been shown to suppress adaptive autophagy and worsen outcomes, and pharmacological inhibition of the related Akt/mTOR pathway has demonstrated harm in preclinical studies [23,24].

Thus, both underfeeding and overfeeding may adversely affect muscle homeostasis [25,26,27,28]. In parallel, mechanical unloading during immobilization induces disuse atrophy, which likely synergizes with systemic catabolic signaling to accelerate muscle wasting [29,30].

Dysfunction is driven by multiple, interrelated biological mechanisms [4]. Recent results from case series and murine models suggest a role for mitochondrial dysfunction in survivors of critical illness with muscle weakness [31,32,33]. Further dysfunctional processes include persistent inflammation with immune cell infiltration into skeletal muscle, potentially sustained by cellular senescence and the senescence-associated secretory phenotype (SASP) [34], which is associated with prolonged muscle weakness and has been documented in ICU survivors for up to nine months [19,35]. Additionally, excitability defects, including ion channel dysfunction and disturbed calcium handling, reduce muscle force independent of muscle mass [14,36]. Microvascular dysfunction with hypoperfusion, typically persisting long beyond normalization of systemic hemodynamics, has also been linked to muscle dysfunction during critical illness [37,38,39]. Finally, impaired muscle regeneration due to muscle satellite cell dysfunction hampers structural recovery [19,40].

### 4.2. Neuropathy

Likewise, ICUAW is not solely a myopathic process but also involves neuropathy. Many of the underlying pathophysiological mechanisms overlap with those observed in myopathy, reflecting the shared vulnerability of peripheral nerves and muscle fibers (Figure 2). At the neuromuscular junction, experimental data from animal models indicate that the expression and clustering of acetylcholine receptors may be downregulated in specific subtypes of ICUAW [14]. At the level of the lower motor neuron and its terminal axon, inflammation with impaired microcirculation and local hypoperfusion of distal axons may induce denervation and axonal degeneration. In this context, the contribution of mechanical compression from interstitial or perineural edema remains controversial, with experimental data providing conflicting evidence [14]. Additionally, reduced motoneuron excitability has been demonstrated in both experimental and clinical studies, resulting in decreased motor output and less muscle force generation [41,42,43].

## 5. Diagnosis

### 5.1. Clinical Assessment

More than three decades after its initial introduction, the Medical Research Council (MRC) Sum Score remains the gold standard for the clinical diagnosis of ICUAW [44]. A dichotomous threshold of 48 points is commonly applied to indicate clinically relevant weakness [45,46]. The MRC Sum Score assesses strength in six bilateral muscle groups (shoulder abduction, elbow flexion, wrist extension, knee flexion, knee extension, and ankle dorsiflexion), yielding a total score ranging from 0 to 60. While pilot trials have provided evidence that classification with a threshold of 48 provides prognostic value, an exploratory analysis of a large cohort of 596 ICU patients suggested the optimal threshold to predict 5-year mortality and disability to be 55—underscoring that even small shifts in clinical classification are associated with meaningful long-term prognostic differences [6,45]. Although the MRC-Sum Score is widely used, reported inter-rater reliability has been heterogeneous across studies, underscoring the importance of structured examiner training and standardized assessment protocols to ensure reproducibility in the ICU setting [46]. Given that bedside strength assessments and monitoring of patient cooperation are often performed within multidisciplinary teams, including nursing staff, consistent application of unified testing procedures is essential. As an alternative to clinical strength assessment, hand-held dynamometry may offer greater sensitivity for detecting subtle changes in muscle strength (cut-off for handgrip strength <11 kg for men and <7 kg for women) [47,48]. Yet, it primarily reflects regional muscle groups—such as grip or quadriceps strength—and may not accurately capture global neuromuscular function in critically ill patients [46,47,49]. Deep tendon reflexes may be diminished in ICUAW [50]; however, due to their limited reproducibility and lack of standardization, they are not routinely incorporated into the clinical grading of ICUAW.

Given the nonspecific clinical presentation of a subacute progressive tetraparesis, careful history-taking and, when indicated, adjunct diagnostic testing is essential to differentiate ICUAW from acute pathologies of the peripheral nervous system [17]. Relevant differential diagnoses include polyradiculoneuropathies (e.g., Guillain–Barré syndrome, acute-onset chronic inflammatory demyelinating polyneuropathy), primary myopathies (infectious or immune-mediated myositis), spinal cord syndromes, and disorders of neuromuscular transmission (e.g., myasthenia gravis or Lambert–Eaton myasthenic syndrome).

Despite its simplicity, non-invasiveness, and feasibility at the bedside, the clinical assessment of muscle strength is often limited in practice due to impaired consciousness and poor patient cooperation. Common confounders include encephalopathy, delirium, and paresis due to acute brain injury. These constraints apply not only to the MRC Sum Score but also to other clinical scoring systems and strength measurements, including instrument-based approaches such as dynamometry.

### 5.2. Electrodiagnostic Testing

Standard electrophysiological protocols combining needle electromyography (EMG) with nerve conduction studies (NCS) allow for the instrumental assessment of neuromuscular involvement in unresponsive or uncooperative patients. Simplified tests, such as the Peroneal Nerve Test (PENT), have been validated as highly sensitive screening tools for ICUAW [51,52]. Comprehensive neurophysiological testing can help rule out rare differential diagnoses and, in cooperative patients, facilitate neurotopographic classification.

A neuropathic component (CIP) typically presents with reduced compound muscle action potentials (CMAPs), while sensory nerve action potentials (SNAPs) are usually less affected, reflecting the predominantly motor nature of the disease [50]. Nerve conduction velocity (NCV) may remain normal or only mildly reduced, as demyelination is uncommon and generally secondary to the axonal pathology [50]. A myopathic pattern also features reduced CMAPs, but without SNAP abnormalities. Spontaneous activity on EMG can be observed in both neurogenic and myogenic processes. In cooperative patients, the analysis of motor unit recruitment patterns can aid in distinguishing between these etiologies or identifying mixed presentations such as CIPNM.

**Figure 3:**
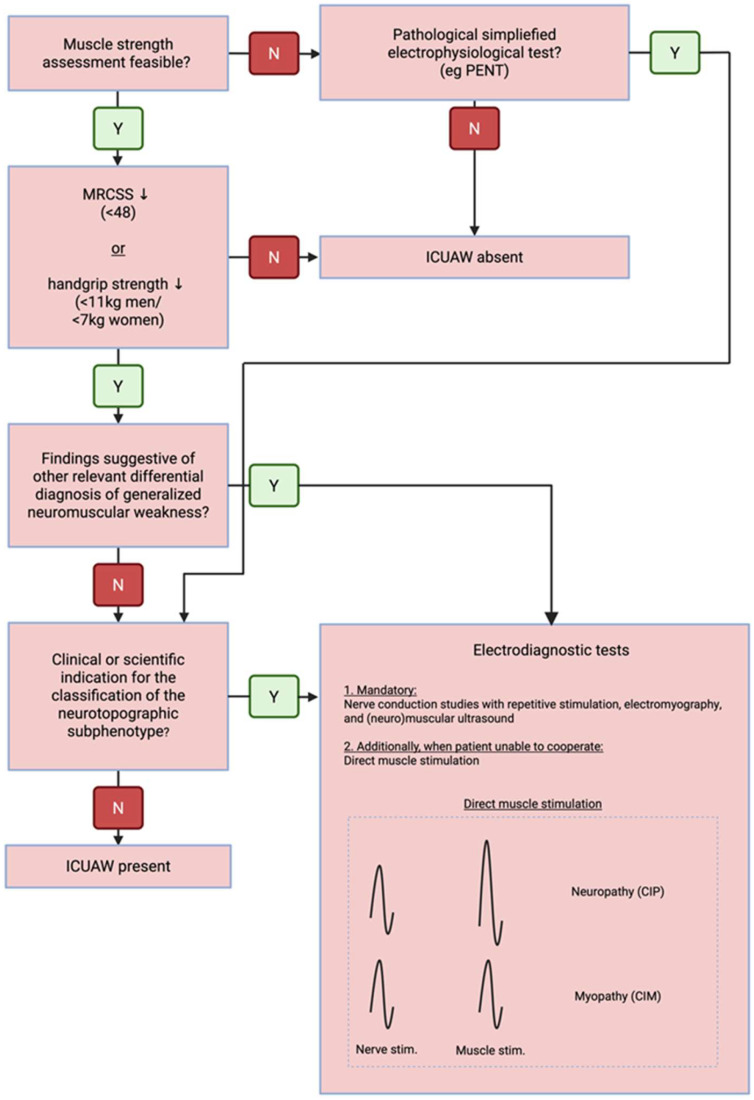
Diagnostic algorithm for ICUAW. The clinical syndromic diagnosis of ICUAW can be made in patients without impaired consciousness through strength testing (MRCSS or dynamometry). However, if a more detailed neurotopographical classification is indicated, or in patients unable to cooperate, additional instrumental diagnostics are required. Since recruitment behavior cannot be assessed in uncooperative patients, direct muscle stimulation is necessary in patients with impaired consciousness to differentiate between CIM and CIP. ICUAW—Intensive Care Unit Acquired Weakness; PENT—Peroneal Nerve Test; CIM—Critical Illness Myopathy; CIP—Critical Illness Polyneuropathy; MRCSS—Medical Research Council Sum Score; Created with Biorender.com.

In unresponsive patients, however, recruitment cannot be assessed, and EMG/NCS alone is insufficient for neurotopographic classification (in both cases, CMAPs are reduced, there may be spontaneous activity either due to denervation or myopathy, and SNAPs are normal in around 40% of CIP cases [50] and are always unremarkable in pure CIM). Hence, direct muscle stimulation to delineate neuropathy from myopathy becomes necessary. Here, a preserved muscle response to direct stimulation supports a diagnosis of CIP over CIM (Figure 3).

The most referenced electrodiagnostic criteria for CIP and CIM by Latronico and Bolton require the presence of both motor and sensory axonal involvement [17]. However, the requirement for abnormal sensory studies in CIP is problematic, as they are often unreliable in critically ill patients and are normal in a substantial subset [50]. For CIM, the demand for muscle biopsy to establish a definite diagnosis limits practical applicability. While these criteria provide a structured framework, they often exceed what is clinically feasible in the ICU setting.

### 5.3. Imaging

In contrast to strength testing, imaging modalities can be employed independently of patient cooperation, although they assess muscle volume as a surrogate rather than directly measuring weakness [13,29]. Among these, ultrasound has emerged as a leading tool, particularly for evaluating the rectus femoris and temporalis muscles [13,29,53,54]. In addition to quantifying muscle atrophy, qualitative analysis of the B-mode image allows inferences about changes in muscle composition [55,56]. Recent developments have explored the use of artificial intelligence (AI) to overcome traditional limitations of ultrasound technology [56]. While CT and MRI offer superior diagnostic resolution, their use as dedicated diagnostic modalities for ICUAW is limited, as they are not suitable for routine implementation outside research contexts. Nevertheless, imaging studies obtained as part of routine clinical care—often performed at heterogeneous time points and across different muscle groups—can frequently be repurposed for diagnostic assessment. Dual-energy X-ray absorptiometry (DXA) for body composition analysis may offer additional information but is hardly applicable in the ICU setting outside of a research context [57].

### 5.4. Diagnostic Algorithm

The diagnostic algorithm for ICUAW should encompass the following key elements, employing the necessary modalities in a stepwise manner and, where appropriate, in a multimodal fashion (Figure 3):

1. Establishment of the syndromic diagnosis of diffuse neuromuscular dysfunction in the context of a preceding critical illness, based on:
  - Clinical findings—symmetric muscle weakness
  - Imaging—diffuse muscle atrophy and reduced muscle quality
  - Electrophysiology—impairment of neuromuscular excitation
2. Exclusion of relevant differential diagnoses
3. Monitoring of neuromuscular dysfunction to support prognostication, prevent secondary complications, and guide therapeutic interventions
4. Phenotypic subclassification using advanced diagnostics, reserved for selected cases where clinically indicated

From a clinical perspective, syndromic-level diagnosis of ICUAW is often sufficient, particularly considering the limited therapeutic options currently available. However, it must be acknowledged that distinct sub-phenotypes exhibit divergent prognoses, thereby justifying further diagnostic refinement during the recovery phase in selected cases.

Yet, from a scientific standpoint, capturing only the final manifestation—namely, muscle weakness—using a binary endpoint (such as MRC-Sum Score  <  48) is inadequate. We argue that stratification based on the underlying pathobiology is essential to advance targeted preventive and therapeutic strategies across the diverse manifestations of ICUAW.

## 6. Prevention and Treatment

By conceptualizing ICUAW within the broader context of multi-organ dysfunction in critical illness, we propose viewing neuromuscular failure as a potentially treatable and treatment-relevant organ dysfunction requiring structured prevention, early recognition, and targeted management in the ICU. At present, however, most effective strategies remain centered on the avoidance or mitigation of established risk factors and on multimodal supportive interventions (Table 1), underscoring the need for more mechanism-driven and phenotype-adapted therapeutic approaches.

### 6.1. Nutrition and Metabolism

The findings from recent large randomized controlled trials (RCTs) in critical care nutrition highlight not only the clinical relevance of this domain but also the necessity of individualized nutritional protocols in the management of critically ill patients [58,59,60]. While caloric and protein deficits exacerbate catabolism and contribute to muscle wasting, overfeeding has also been linked to increased mortality and impaired muscle function [23,25,26,28,61,62,63,64,65]. The latter is explained pathophysiologically by mechanisms such as anabolic resistance (reduced uptake of supplemented nutrients during the catabolic phase) and suppression of adaptive metabolic responses (such as ketogenesis and autophagy) [14,58]. Attempts to pharmacologically mitigate anabolic resistance using agents such as anabolic steroids (e.g., oxandrolone), growth hormone, or propranolol, as well as interventions such as glutamine supplementation or intravenous immunoglobulin therapy, have failed to demonstrate meaningful efficacy in the context of ICUAW [66]. Furthermore, isocaloric feeding regimens that fail to account for endogenously mobilized substrates during early critical illness can worsen stress-induced hyperglycemia [67]. Given these pathophysiological insights, three metabolically modifiable variables emerge as central to ICUAW: caloric intake, protein intake, and glycemic control.

**Figure 4:**
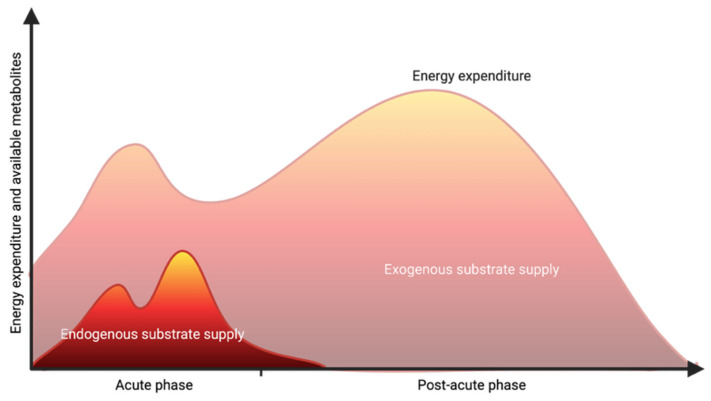
Metabolism during critical illness. Created with Biorender.com.

Accurate estimation of macronutrient requirements, particularly caloric and protein needs, hinges on reliable determination of the resting energy expenditure (REE). Current international guidelines endorse bedside indirect calorimetry as the gold standard for REE assessment [68,69,70]. If indirect calorimetry is unavailable, the Harris–Benedict equation is preferred over other predictive formulas [71]. Moreover, in light of the risks associated with overfeeding, it is advisable to tailor energy provision and protein targets not only to static variables such as REE, but also to the patient’s individual metabolic tolerance. As endogenous substrate mobilization contributes substantially to total energy availability in phases of critical illness, matching exogenous intake 1:1 to REE may result in overfeeding overall (Figure 4). Although no validated standard exists, this metabolic tolerance is commonly assessed using time-based strategies (e.g., acute vs. post-acute phase) or metabolic biomarkers, such as insulin requirements or serum phosphate levels [70]. European guidelines follow a time-based approach and recommend initiating low-dose enteral nutrition within 48 h of ICU admission, aiming for 70% of caloric and protein targets, with gradual escalation over the following 3–7 days. When indirect calorimetry is employed, the goal is to reach 80–100% of measured REE by day 7; if predictive equations are used, a target of 70% of REE is advised [68]. The German guideline also incorporates persistent hyperglycemia with elevated insulin requirements or declining serum phosphate levels as indicators of reduced metabolic tolerance and suggests temporary reduction of energy and protein provision (mean insulin 2–4 IU/h—50% of REE/protein target; mean insulin requirements >4 IU/h or serum phosphate <0.65 mmol/L—25% of REE/protein target) under these conditions [70].

Regarding optimal protein supplementation, observational studies have suggested a positive correlation between higher protein intake and improved clinical outcomes [72,73,74]. However, recent RCTs have challenged this assumption. The PRECISe trial, which included 935 ventilated patients, found that higher protein dosing (2.0 g/kg/day vs. 1.3 g/kg/day) was associated with worse health-related quality of life at 180 days (primary outcome measure) [75]. Similarly, the EFFORT Protein Study (*n* = 1329) did not demonstrate a survival benefit with high protein intake (≥2.2 g/kg/day vs. ≤1.2 g/kg/day), and even reported increased mortality among patients with acute kidney injury [76]. Finally, the cluster-randomized TARGET Protein trial likewise showed no benefit from high-protein enteral formulas (days free of the index hospital and alive at day 90: 62 (0–77) versus 64 (0–77) days, median difference −1.97; 95% CI −7.24 to 3.30; *p* = 0.46). This result was consistent over all secondary endpoints [77]. A recent meta-analysis confirmed the lack of general benefit from elevated protein dosing and suggested potential harm in the context of renal dysfunction [78]. In alignment with these findings, current European guidelines recommend a daily protein intake of 1.3 g/kg body weight during the ICU stay [68].

The optimal target range for blood glucose in critically ill patients has been the subject of numerous large RCTs [67,79,80,81]. While earlier studies demonstrated that strict glycemic control conferred benefits in terms of morbidity and mortality—particularly in patients receiving early parenteral nutrition [67,81]—the more recent TGC-fast trial found no survival advantage for intensive glycemic control in patients not receiving early parenteral nutrition (odds ratio 1.00 [0.96–1.04], 80–110 mg/dL vs. <215 mg/dL) [79]. A similar discrepancy emerges in the context of ICUAW: post hoc analyses of the Leuven I & II trials suggested a reduction in ICUAW incidence with intensive insulin therapy [67,81]. However, in the modern TGC-fast trial, surrogate outcomes of ICUAW such as duration of mechanical ventilation and ICU length of stay were not significantly different [79]. A key difference between the earlier and more recent trials is the lower severity of hyperglycemia observed in the TGC-fast cohort, which may be attributed to modern nutritional strategies—namely, hypocaloric feeding during the acute phase and the avoidance of early parenteral nutrition. In light of these findings, current guidelines continue to endorse a more liberal approach to glycemic control, with a recommended target range of 140–180 mg/dL [82,83,84].

### 6.2. Drug Exposure

A number of medications have been associated with an increased risk of ICUAW in observational studies (see Table 1), including glucocorticoids [85,86,87], antibiotics such as aminoglycosides or vancomycin [88,89,90], and vasopressors [91]. However, given the overall low quality and heterogeneity of the available evidence—alongside the frequent clinical necessity of these agents—the authors do not consider these associations to warrant specific therapeutic consequences at present. Regarding neuromuscular blocking agents, observational data have suggested a possible association with increased ICUAW incidence [88,92]. However, no significant difference in muscle strength has been observed between cisatracurium and placebo in RCTs conducted in deeply sedated acute respiratory distress (ARDS) patients [93]. A subsequent RCT comparing deep sedation with neuromuscular blockade versus light sedation without paralytics actually reported higher rates of ICUAW in the group receiving both sedation and neuromuscular blockade [94]. These findings suggest that neuromuscular inactivity, rather than any specific pharmacologic effect of relaxants, may be the primary driver of ICUAW in this context.

### 6.3. Mobilization and Neuromuscular Electrical Stimulation

The role of neuromuscular inactivity as a modifiable therapeutic target is further underscored by evidence from studies on (early) mobilization and electrical stimulation techniques [30,95,96,97,98,99].

Here, several landmark trials provided evidence that early (<72 h) and protocol-based mobilization is associated with favourable outcomes after ICU treatment. Specifically, RCTs have shown that initiating mobilisation within 72 h of mechanical ventilation is associated with improved outcomes, including greater functional independence, mobility, shorter ICU and hospital stays, more delirium- and ventilation-free days, higher likelihood of discharge home, and long-term cognitive and functional benefits [100,101,102]. In contrast, studies where mobilisation was started at later time points found no such positive effects [103,104]. These effects were also consistent over several meta-analyses [105,106]. Furthermore, implementing mobilisation protocols has been proven to enhance feasibility, safety, and duration, as well as the level of mobilisation [107,108].

There are also multiple levels of evidence to support the role of active versus passive mobilisation. In a recent prospective study from our group in a neurocritical care cohort, objectively measured active movement was an independent predictor of muscle atrophy during the ICU stay [29]. This is in line with negative results from a recent RCT on (primarily passive) bed-cycling in ventilated patients [109]. Also, several clinical trials investigating electrical muscle stimulation (EMS) as a principle to promote muscle activation showed overall positive effects on physical outcomes in the ICU population [95,110]. However, the TEAM trial, using early active mobilization in mechanically ventilated patients at the highest level possible, found no significant differences compared to usual care in days alive and out of hospital at 180 days [96]. Importantly, these findings should not be interpreted as evidence against the general effectiveness of active mobilization strategies. Rather, they highlight important limitations of the study, such as insufficient treatment separation and the relatively short duration of the intervention, which was limited to the ICU stay.

While early mobilization is a central therapeutic principle to limit ICUAW, its implementation is often limited by patient non-cooperation and the inherent risks of mobilizing critically ill individuals. A review estimated the rate of adverse events at 2.6%, and the rate of serious adverse events at 0.6% [111]. This underscores the need for complementary strategies to support neuromuscular recovery. Hence, enhancing activity via EMS in the absence of mobility may be a viable strategy in patients at high risk of complications during mobilization or with limited capacity for active participation.

## 7. Conclusions

Given the diverse pathophysiological mechanisms and neurotopographic diagnoses underlying ICUAW, the implementation of a standardized therapeutic bundle (analogous to established approaches for other critical care syndromes such as delirium) appears warranted. At the same time, advancing the biological definition of ICUAW is essential to enable more precise, goal-directed therapeutic strategies. While multimodal intervention bundles provide a necessary foundational framework, future care will likely require personalization within these structures—guided by phenotypes, biomarkers, and patient-specific risk profiles. Interventional studies evaluating such multimodal strategies, as well as the systematic inclusion of ICUAW as a clinically relevant endpoint in critical care trials, are essential to advance the evidence-based management of this common complication. Moreover, a longitudinal care strategy extending beyond the ICU stay is crucial and should involve all relevant levels of the healthcare system. Preventive and therapeutic strategies initiated in the ICU should be viewed as the starting point of a longitudinal rehabilitation trajectory. Continuity of nutritional assessment and progressive mobilization after ICU discharge is critical to sustain recovery and mitigate persistent neuromuscular impairment. From both a scientific and clinical standpoint, we therefore also advocate for the development of dedicated outpatient follow-up programs, such as specialized PICS clinics, to ensure comprehensive aftercare. These would provide the necessary interdisciplinary expertise to address the complexity of ICUAW and support long-term recovery.

## Figures and Tables

**Table 1:** Modifiable and non-modifiable risk factors of ICUAW. Asterisks (*) indicate modifiable factors that are largely influenced by multidisciplinary ICU care, thereby representing potential targets for preventive interventions.

Modifiable Risk Factors	Non-Modifiable Risk Factors
Hyperglycemia *	Age
Catabolism/Underfeeding *	Female sex
Overfeeding *	Sarcopenia/Frailty
Vasopressors	Disease severity
Sedatives and neuromuscular blockers	
Aminoglycosides, vancomycin	
Inactivity *	

Adapted from Vanhorebeek et al. [10].

## Data Availability

Data sharing is not applicable to this article as no new data were created or analyzed in this study.
